# Tracing the Origin and Northward Dissemination Dynamics of HIV-1 Subtype C in Brazil

**DOI:** 10.1371/journal.pone.0074072

**Published:** 2013-09-12

**Authors:** Edson Delatorre, José C. Couto-Fernandez, Monick Lindenmayer Guimarães, Ludimila Paula Vaz Cardoso, Keila Correia de Alcantara, Mariane Martins de Araújo Stefani, Hector Romero, Caio C. M. Freire, Atila Iamarino, Paolo M. de A Zanotto, Mariza G. Morgado, Gonzalo Bello

**Affiliations:** 1 Laboratório de AIDS e Imunologia Molecular, Instituto Oswaldo Cruz, FIOCRUZ, Rio de Janeiro, Brazil; 2 Instituto de Patologia Tropical e Saúde Pública, Universidade Federal de Goiás, Goiânia, Brazil; 3 Laboratorio de Organización y Evolución del Genoma, Sección Biomatemáticas, Facultad de Ciencias, Universidad de la República, Montevideo, Uruguay; 4 Laboratório de Evolução Molecular e Bioinformática, Departamento de Microbiologia, Instituto de Ciências Biomédicas, Universidade de São Paulo, São Paulo, Brazil; Chinese Academy of Sciences, Wuhan Institute of Virology, China

## Abstract

Previous studies indicate that the HIV-1 subtype C epidemic in southern Brazil was initiated by the introduction of a single founder strain probably originating from east Africa. However, the exact country of origin of such a founder strain as well as the origin of the subtype C viruses detected outside the Brazilian southern region remains unknown. HIV-1 subtype C *pol* sequences isolated in the southern, southeastern and central-western Brazilian regions (*n* = 209) were compared with a large number (*n* ~ 2,000) of subtype C *pol* sequences of African origin. Maximum-likelihood analyses revealed that most HIV-1 subtype C Brazilian sequences branched in a single monophyletic clade (C_BR-I_), nested within a larger monophyletic lineage characteristic of east Africa. Bayesian analyses indicate that the C_BR-I_ clade most probably originated in Burundi and was introduced into the Paraná state (southern region) around the middle 1970s, after which it rapidly disseminated to neighboring regions. The states of Paraná and Santa *Catarina* have been the most important hubs of subtype C dissemination, and routine travel and spatial accessibility seems to have been the major driving forces of this process. Five additional introductions of HIV-1 subtype C strains probably originated in eastern (*n* = 2), southern (*n* = 2) and central (*n* = 1) African countries were detected in the Rio de Janeiro state (southeastern region). These results indicate a continuous influx of HIV-1 subtype C strains of African origin into Brazil and also unveil the existence of unrecognized transmission networks linking this country to east Africa.

## Introduction

The global spread of the Human immunodeficiency virus type 1 (HIV-1) group M that took place in the second half of the twentieth century was associated to the random exportation of some viral strains out of the epicenter in Central Africa into previously unexposed human populations [1]. The subsequent dissemination and diversification of the virus within such populations has resulted in the differential global distribution of HIV-1 group M subtypes and inter-subtype recombinants. The most prevalent HIV-1 group M variant worldwide is subtype C, which accounts for nearly half (48%) of all global infections [2]. This HIV-1 subtype is particularly prevalent in several countries from southern, eastern and central Africa, India and Brazil.

The Brazilian HIV-1 subtype C epidemic has been mostly restricted to the states of the southern region (Rio Grande do Sul [RS], Santa Catarina [SC] and Paraná [PR]) where this subtype accounts for between 20% and 80% of HIV-1 infections [3]. Previous phylogeographic studies indicate that the subtype C epidemic in southern Brazil was probably initiated by the introduction of a single founder strain into PR, followed by a rapid dissemination of the virus to the neighboring southern states [4,5]. The founder Brazilian subtype C strain probably originated in east Africa, although the exact country of origin and the precise time-scale of such an event remain uncertain [6]. One study conducted by our group points to Burundi as the most probable origin of the Brazilian subtype C clade [4], while other studies point to Ethiopia or Kenya [5,7]. Initial estimates based on viral strains mostly sampled in Rio Grande do Sul propose that the founder event occurred around the early 1980s [4,8], but another study based on samples from several states traced back the origin of the Brazilian subtype C epidemic to 1960-1970 [5].

Recent studies have also documented a significant proportion of HIV-1 subtype C infections among individuals from different states across the southeast, central-west and north Brazilian regions, supporting a northward spread of HIV-1 subtype C in Brazil [3]. Subtype C was observed in 6-8% of patients from the São Paulo (SP) state [9,10], 0.5-1% of patients from the Rio de Janeiro (RJ) state [11,12,13], 3-11% of patients from the Goiás (GO) state [14,15,16], 5% of patients from the Mato Grosso (MT) state [17], 10% of patients from the Mato Grosso do Sul (MS) state [18] and 6% of patients from the Tocantins state [19]. Although those studies support an influx of variants from the southern region, the exact origin and dissemination dynamics of Brazilian subtype C viruses circulating outside the southern states has not been studied in detail up to date.

In the present study, we used a comprehensive data set of Brazilian (*n* = 209) and African (*n* > 2,000) HIV-1 subtype C *pol* sequences to reconstruct with more precision the geographic origin and the onset date of the HIV-1 subtype C clade introduced into southern Brazil. Moreover, we traced the dissemination dynamics of the HIV-1 subtype C epidemic in the southeast and central-west Brazilian regions. Spatial and temporal information were combined in a Bayesian framework to reconstruct migration events both within Brazil and between African countries and Brazil.

## Materials and Methods

### HIV-1 subtype C Brazilian sequences

**Figure 1:**
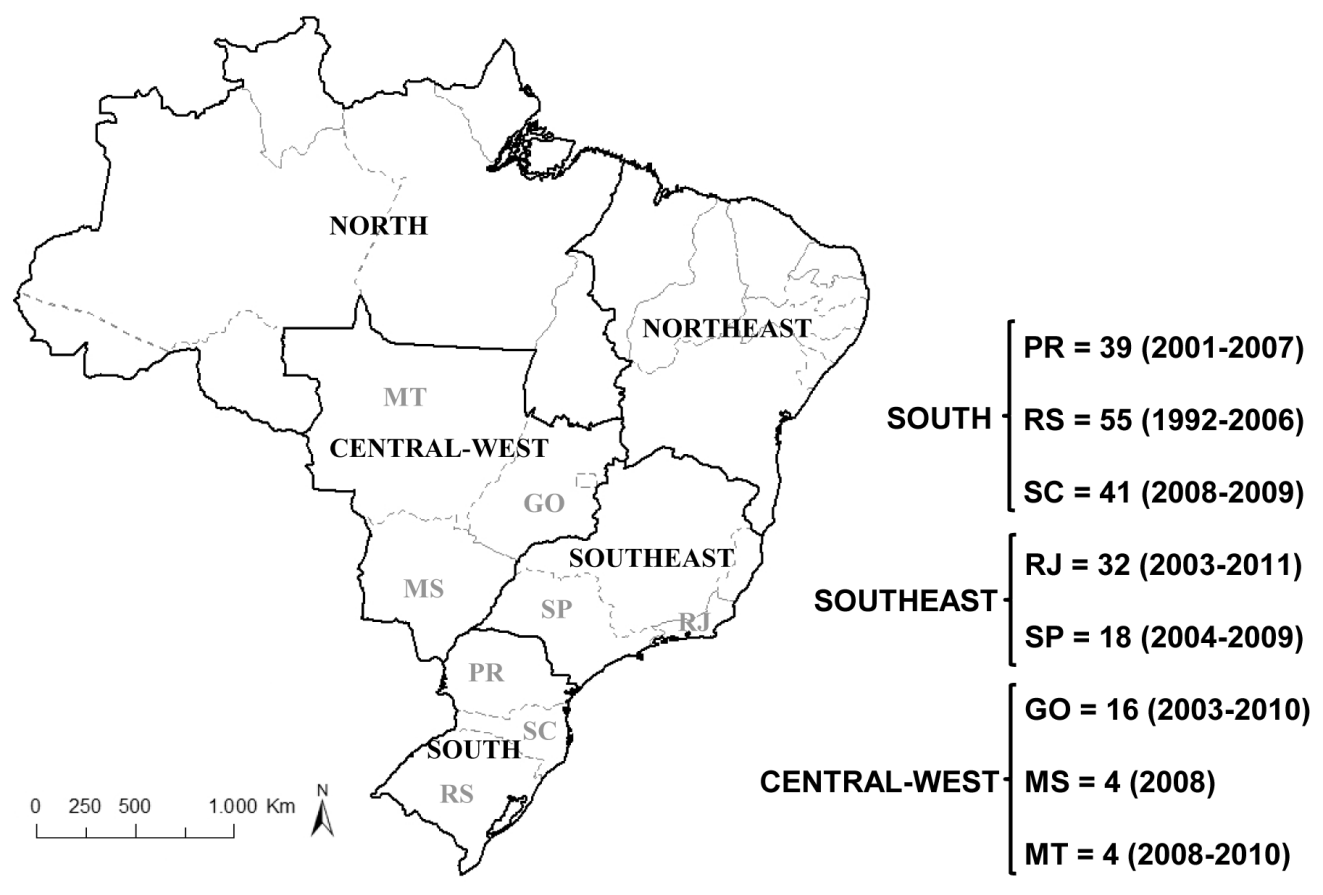
Political map of Brazil showing the country regions and states. Boundaries of regions and states are indicated by black and gray lines, respectively. The position of the eight states analyzed in the present study is indicated with a two letter code: GO (Goiás), MT (Mato Grosso), MS (Mato Grosso do Sul), PR (Paraná), RJ (Rio de Janeiro), RS (Rio Grande do Sul), SC (Santa Catarina) and SP (São Paulo). The number and sampling dates of HIV-1 subtype C pol sequences from each location included in the present study are indicated.

The Brazilian HIV-1 subtype C dataset was composed of 209 sequences covering the entire protease and partial reverse transcriptase (PR/RT) genes (nt 2253-3272 relative to HXB2 clone) collected in eight states from the south (RS, SC and PR), southeast (SP and RJ), and central-west (GO, MT and MS) regions of Brazil (Figure 1). New PR/RT subtype C sequences were obtained from 32 individuals from RJ selected from a larger cohort of about 3,000 HIV-infected patients followed at outpatient clinics from the Public Health System distributed throughout the state that underwent HIV genotyping tests at the Laboratory of AIDS and Molecular Immunology (FIOCRUZ) between 2002 and 2011, as previously described [20]. The HIV-1 subtype C *pol* sequences from RJ were combined with sequences from SP (*n* = 18), GO (*n* = 16), MT (*n* = 4) and MS (*n* = 4) available at the Los Álamos HIV Sequence Database (www.hiv.lanl.gov) and described elsewhere [10,14,15,16,17,18,21,22,23,24], and with a dataset of sequences isolated in the south region (RS = 55, SC = 41 and PR = 39) described in detail in a previous study [25]. The study was approved by the Instituto Oswaldo Cruz - Ethics Committee. No informed consent from participants was obtained as the data were analyzed anonymously.

### HIV-1 subtype C reference dataset

The HIV-1 subtype C Brazilian sequences were initially aligned with a reference set of 1,961 subtype C *pol* gene sequences of African origin obtained from the Los Álamos HIV Sequence Database. This reference data set, described in more detail in our previous study [26], includes subtype C sequences from therapy-naïve patients representative of the east (Burundi, Ethiopia, Kenya, Tanzania and Uganda), southern (Botswana, Malawi, Mozambique, South Africa, Zambia and Zimbabwe) and central (Angola and Democratic Republic of Congo) African regions sampled over a time period of 25 years (1986-2010). The basic local alignment search tool (BLAST) (www.ncbi.nlm.nih.gov/BLAST) was also used to select 50 subtype C reference sequences with known sampling dates isolated world-wide that displayed a high similarity score (> 95%) to specific Brazilian subtype C strains. The subtype assignment of all sequences here included was confirmed using the REGA HIV subtyping tool v.2 [27].

### Sequence alignment and analysis of phylogenetic signal

Sequences were aligned using the CLUSTAL X program [28]. To avoid any bias on the phylogenetic reconstructions, all sites with major antiretroviral drug resistance mutations in PR (50, 82 and 90) and RT (41, 67, 70, 98, 103, 106, 179, 184, 190, 215 and 219) detected in at least two sequences were excluded from those alignments containing Brazilian subtype C sequences retrieved from treated-patients. All alignments are available from the authors upon request. Substitution saturation was evaluated in each alignment by plotting the estimated number of transitions and transversions against genetic distance for each pairwise comparison, using the DAMBE program [29]. The phylogenetic signal in each alignment was also investigated with the likelihood mapping method [30] by analyzing 10,000 random quartets. Likelihood mapping analyses were performed with the TREE-PUZZLE program [31], using the online web platform Phylemon 2.0 [32].

### Phylogenetic analysis

Maximum Likelihood (ML) phylogenetic trees were inferred under the GTR+I+Γ_4_ nucleotide substitution model, selected using the jModeltest program [33]. The ML tree was reconstructed with the PhyML program [34] using an online web server [35]. Heuristic tree search was performed using the SPR branch-swapping algorithm and the reliability of the obtained topology was estimated with the approximate likelihood-ratio test (aLRT) [36] based on the Shimodaira-Hasegawa-like procedure. The ML trees were visualized using the FigTree v1.3.1 program [37].

### Analysis of spatiotemporal dispersion pattern

The evolutionary rate (µ, nucleotide substitutions per site per year, subst./site/year), the age of the most recent common ancestor (*T*_mrca,_ years), and the spatial dynamics of different HIV-1 subtype C clades were jointly estimated using the Bayesian Markov Chain Monte Carlo (MCMC) approach as implemented in BEAST v1.7.4 [38,39]. Analyses were performed using the GTR+I+Γ_4_ nucleotide substitution model, an uncorrelated Lognormal relaxed molecular clock model [40] and a Bayesian Skyline coalescent tree prior [41]. Migration events and the most relevant migration pathways between locations were identified by applying a standard discrete Bayesian phylogeographic model and the Bayesian stochastic search variable selection (BSSVS) approach [42], respectively. Migratory events and significant non-zero rates were summarized using the cross-platform SPREAD application [43] and viewed with Google, Earth (http://earth.google.com). MCMC chains were run for 4-5 × 10^8^ generations and adequate chain mixing was checked, after excluding an initial 10%, by calculating the effective sample size (ESS) using the TRACER v1.5 program [44]. Maximum clade credibility (MCC) trees were summarized from the posterior set of trees (PST) with TreeAnnotator and visualized with FigTree v1.3.1.

Viral exchange rates among localities in Brazil were also estimated as transition rates between discrete characters along a PST generated using MrBayes v3.2.1 [45]. The PST was obtained during MCMC convergence from two independent runs with 2 × 10^7^ generations and sampled at each 2,000 generations, employing the GTR+I+Γ_4_ substitution model. Transition rates (q) were estimated using the APE package v3.06 [46] implemented in the R statistical environment v2.15.2 [47], under three different models: completely asymmetrical (ARD), symmetrical (SYM) and equal rates (ER). The best-fit model to our data was chosen by the comparison of the marginal Likelihoods from each one after 10,000 bootstrap replications, using the method proposed by Suchard et al. [48] implemented in Tracer v1.5. Due to the great uncertainty on the phylogenetic topologies obtained from HIV sequences and given that the estimated *q* values are subject to this issue, outliers from *q*’ s distribution were removed using the boxplot function in R.

### GenBank accession numbers

Nucleotide sequences obtained during our study have been assigned GenBank accession numbers KF247210, KF255836-KF255866.

## Results

### Identification of multiple HIV-1 subtype C introductions in Brazil

**Figure 2:**
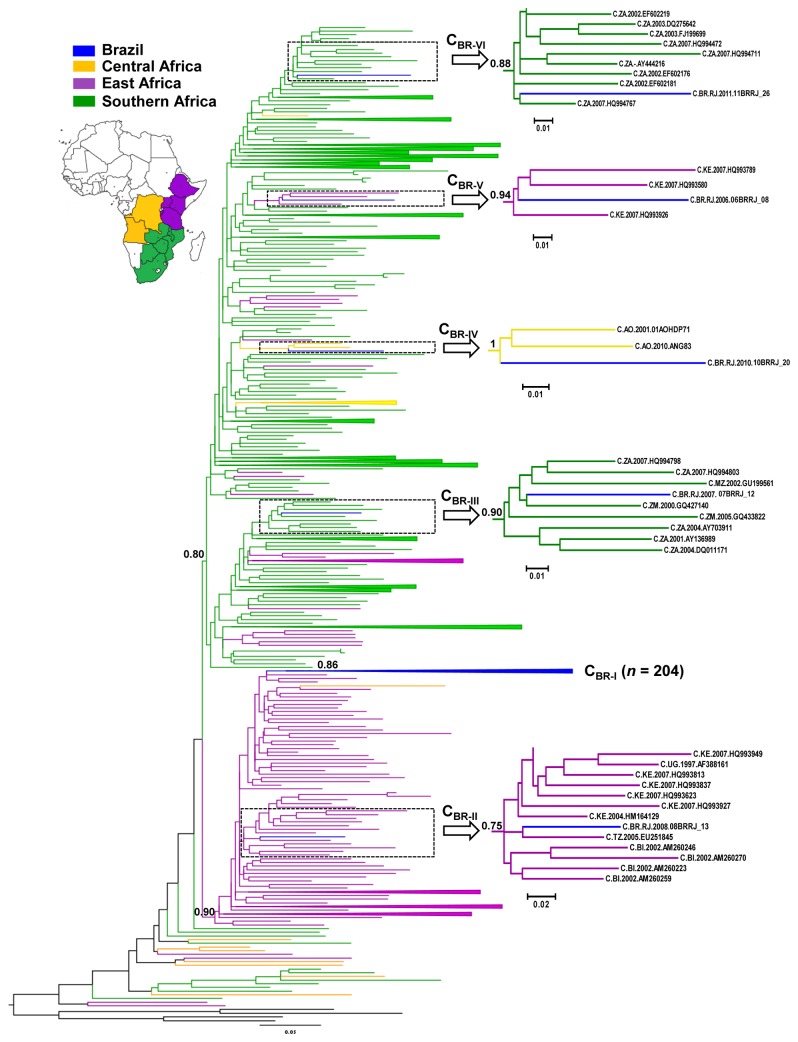
ML tree of HIV-1 subtype C pol (~1,000 bp) sequences from Brazil (*n* = 209) and the central (*n* = 53), eastern (*n* = 332) and southern (*n* = 645) African regions. The color of the branches represents the geographic region from where the subtype C sequences originated, according to the legend and map provide in the figure. The dotted boxes highlight the position of the Brazilian subtype C lineages (C_BR-II_ to C_BR-IV_) that branched outside the major Brazilian clade (C_BR-I_). A close view of the minor Brazilian subtype C lineages and the most closely related African sequences is also provided. For visual clarity, the Brazilian clade C_BR-I_ and some clades that comprised mostly sequences from central, eastern or southern Africa were collapsed into triangles. The *aLRT* support values are indicated only at key nodes. The tree was rooted using HIV-1 subtype A1 and D reference sequences (black branches). Horizontal branch lengths are drawn to scale with the bar at the bottom indicating nucleotide substitutions per site.

Four different datasets were used to reconstruct the origin and spatiotemporal dynamics of HIV-1 subtype C in Brazil (Tables S1 to S4). The transition/transversion *vs* divergence graphics and the likelihood-mapping analyses showed that all HIV-1 subtype C *pol* datasets used in this study contain enough evolutionary information for reliable phylogenetic and molecular clock inferences (Figure S1). The first dataset, here called C_AFR+BR_ (Table S1), was used to characterize the relationship between viruses sampled in Brazil (*n* = 209) with those circulating at 13 African countries (*n* = 1,961) with an estimated subtype C prevalence >5% [2]. The Brazilian subtype C strains were initially compared with those sequences from South Africa that represent the majority (52%) of subtype C sequences in our dataset. The close relative South African sequences were selected up to a maximum of 100 (Figure S2) and combined with subtype C sequences from the other African countries. The final ML phylogenetic tree revealed that most (98%) subtype C sequences from Brazil branched within a single monophyletic cluster (C_BR-I,_ aLRT = 0.86) that was nested within a highly supported subtype C monophyletic clade (C_EA,_ aLRT = 0.90) (Figure 2). The C_EA_ clade has been previously associated to the east African region [26] and comprise 73% of sequences from east Africa, 9% of sequences from central Africa, and none of sequences from southern Africa included in this analysis. Five (2%) Brazilian subtype C sequences branched outside the C_BR-I_ clade, constituting independent lineages. The lineage C_BR-II_ branched within the C_EA_ clade, while the remaining four lineages (C_BR-III_ to C_BR-VI_) were dispersed outside that clade (Figure 2). All Brazilian subtype C sequences that branched outside the C_BR-I_ clade were sampled from Brazilian individuals who live in RJ state and were diagnosed with HIV-infection between 2006 and 2011.

### Origin of Brazilian HIV-1 subtype C clades

**Figure 3:**
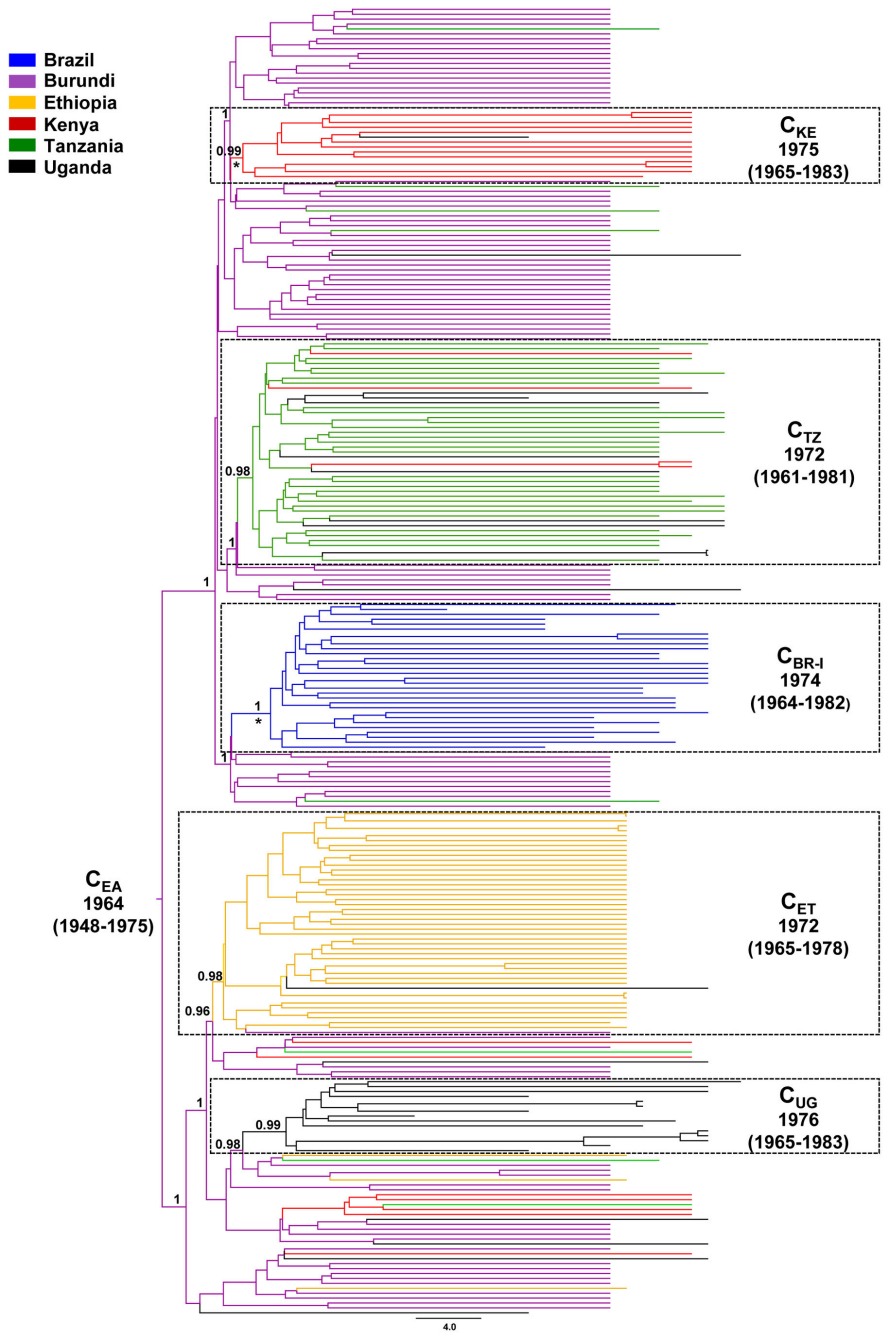
Time-scaled Bayesian MCC tree of the HIV-1 C_EA_ and C_BR-I_ lineages. Branches are colored according to the most probable location state of their descendent nodes. The legend for the colors is shown on the left. The dotted boxes highlight the position of the major country-specific sub-clades detected in our study. The median age (with 95% HPD interval in parentheses) and *PSP* values of key nodes are shown. Asterisks point to key nodes with a high (> 0.85) *PP* support. Horizontal branch lengths are drawn to scale with the bar at the bottom indicating years. The tree was automatically rooted under the assumption of a relaxed molecular clock.

The origin of each Brazilian HIV-1 subtype C clade was reconstructed using a Bayesian statistical framework that allows ancestral reconstruction of the locations at the interior nodes of Bayesian trees while accommodating phylogenetic uncertainty. To trace the origin of the C_BR-I_ clade we used a dataset (C_EA+BR-I_) that combines all sequences from east Africa (*n* = 236) that branched within the C_EA_ clade and a subset of Brazilian sequences (*n* = 30) that were representative of the C_BR-I_ lineage (Table S2). The median evolutionary rate of the C_EA+BR-I_ *pol* dataset, estimated under a chronological time-scale employing the dates of the sequences, was 1.8 × 10^-3^ (95% highest posterior density [HPD]: 1.3 × 10^-3^ - 2.4 × 10^-3^) subst./site/year. The Bayesian MCC tree indicates that the C_BR-I_ clade most probably originated in Burundi (posterior state probability, PSP = 1) at around the middle 1970s, coinciding with the emergence of other major country-specific subclades in several east African countries including: Ethiopia (C_ET_), Kenya (C_KE_), Tanzania (C_TZ_) and Uganda (C_UG_) (Figure 3).

**Figure 4:**
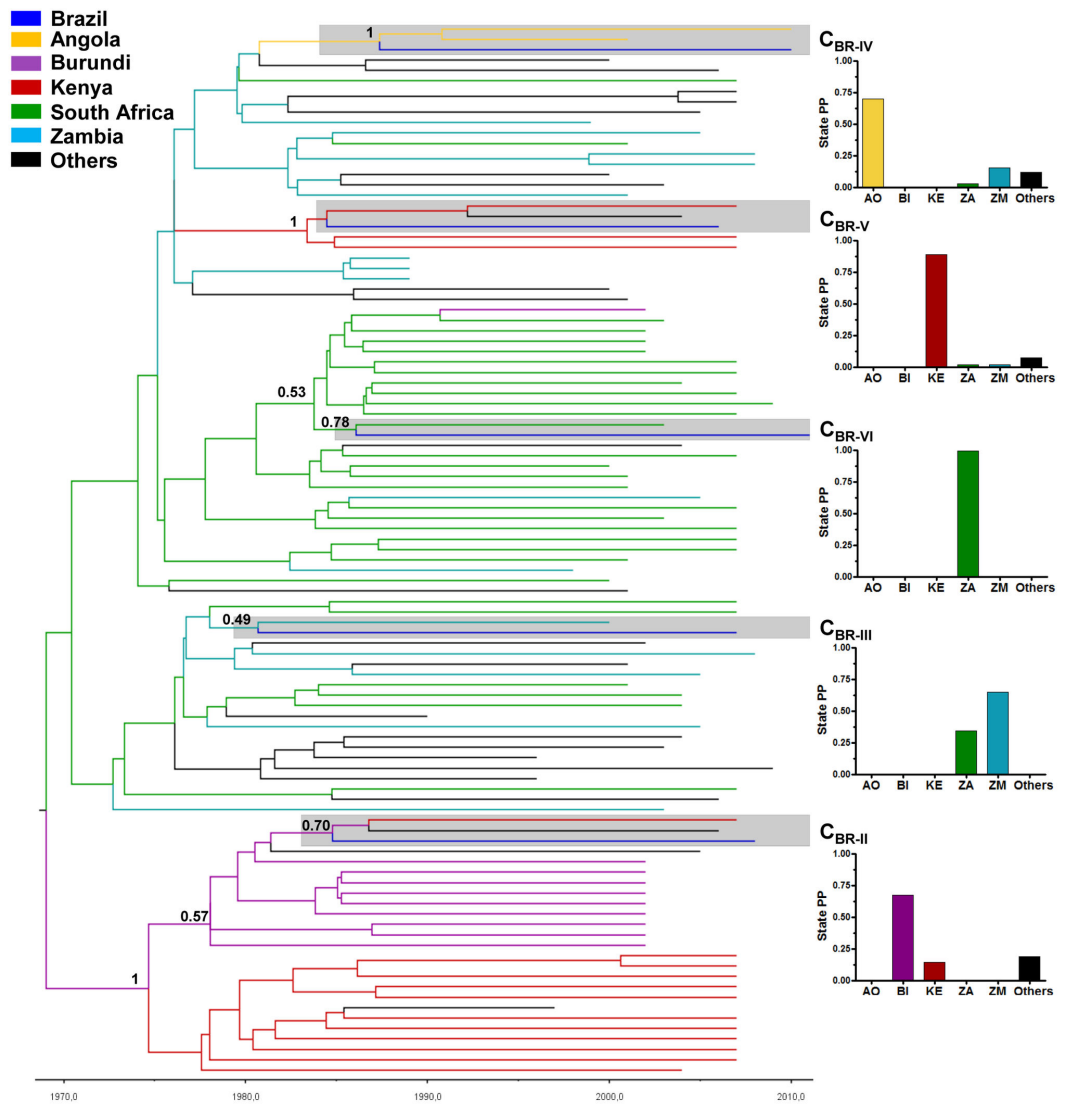
Time-scaled Bayesian MCC tree of the HIV-1 C_BR-II_ to C_BR-VI_ lineages and the most closely related reference sequences. Branches are colored according to the most probable location state of their descendent nodes. The legend for the colors is shown on the left. The boxes highlight the position of the minor Brazilian HIV-1 subtype C clades. The *PP* support is indicated only at key nodes. The scale bar at the bottom indicates years. The tree was automatically rooted under the assumption of a relaxed molecular clock. Graphics on the right depict the *PSP* distributions at the first ancestral nodes of Brazilian subtype C lineages at the Bayesian MCC tree. Countries represented are AO (Angola), BI (Burundi), KE (Kenya), ZA (South Africa), ZM (Zambia) and others (from Asia and Europe).

To determine the most probable geographic origin of the minor Brazilian subtype C clades we used an independent dataset (C_AFR+BR-II-VI_) that combines the sequences C_BR-II_ to C_BR-IV,_ their closest relative African sequences that branched with each minor Brazilian clade until the second ancestral node in the ML phylogenetic tree, and those subtype C sequences isolated world-wide with the highest BLAST search similarity score (> 95%) to each of the minor Brazilian subtype C lineages (Table S3). The Bayesian MCC tree suggests that the C_BR-II_ clade most probably originated in Burundi (PSP = 0.67) or Kenya (PSP = 0.14), the C_BR-III_ clade in Zambia (PSP = 0.65) or South Africa (PSP = 0.34), the C_BR-IV_ clade in Angola (PSP = 0.70) or Zambia (PSP = 0.15), the C_BR-V_ clade in Kenya (PSP = 0.89) and the C_BR-VI_ clade in South Africa (PSP = 0.99) (Figure 4).

### Spatiotemporal dispersal pattern of the HIV-1 C_BR-I_ clade

**Figure 5:**
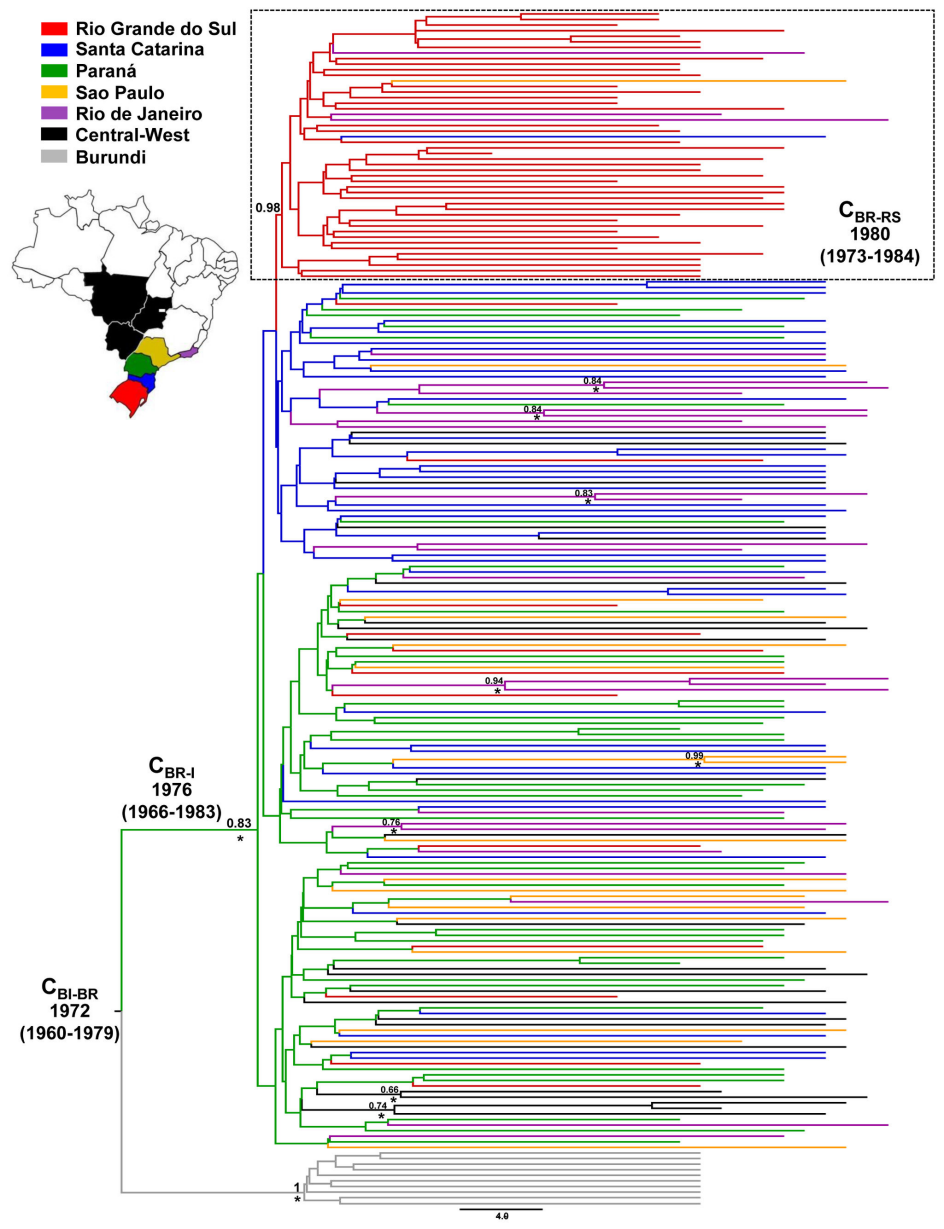
Time-scaled Bayesian MCC tree of the HIV-1 C_BR-I_ lineage. Branches are colored according to the most probable location state of their descendent nodes as indicated in the legend and map shown on the left. The dotted boxes highlight the position of the Brazilian sub-clade characteristic of Rio Grande do Sul (C_BR-RS_). The median age (with 95% HPD interval in parentheses) and the *PSP* values of some key nodes are shown. Key nodes with a high (> 0.85) *PP* support are marked with an asterisk. Horizontal branch lengths are drawn to scale with the bar at the bottom indicating years. The tree was automatically rooted under the assumption of a relaxed molecular clock.

To reconstruct the spatiotemporal dynamics of dissemination of the major Brazilian clade we used a fourth dataset (C_BI+BR-I_) that comprises all Brazilian subtype C sequences that branched within the C_BR-I_ clade and a subset of 10 closely related sequences from Burundi (Table S2). The median evolutionary rate for this subtype C *pol* dataset also estimated under a chronological time-scale employing the dates of the sequences was 2.0 × 10^-3^ (95% HPD: 1.4 × 10^-3^ - 2.6 × 10^-3^) subst./site/year. The Bayesian analysis placed the most probable root location in the state of PR (PSP = 0.83), followed by SC (PSP = 0.15), and set the maximum and minimum dates for such a founder event to 1972 (median *T*_mrca_ of the Brazilian and the closest Burundian sequences) and 1976 (median *T*_mrca_ of the C_BR-I_ clade), respectively (Figure 5). The overall topology of the Bayesian phylogenetic tree showed a great level of phylogenetic intermixing of Brazilian subtype C sequences from different geographic locations, with the exception of RS. A high proportion (78%) of subtype C sequences from RS branched within a single state-specific monophyletic cluster (C_BR-RS_) (Figure 5). This analysis also identifies seven highly supported (posterior probability, *PP* > 0.85) geographic-specific monophyletic clades of small size (2-3 sequences) outside the southern region (RJ = 4, SP = 1, GO/MT = 1 and GO/MS = 1) (Figure 5). These local clusters comprise 11 (35%) out of 31 subtype C sequences from RJ, 2 (11%) out of 18 sequences from SP and 5 (21%) out of 24 sequences from the central-west region.

**Figure 6:**
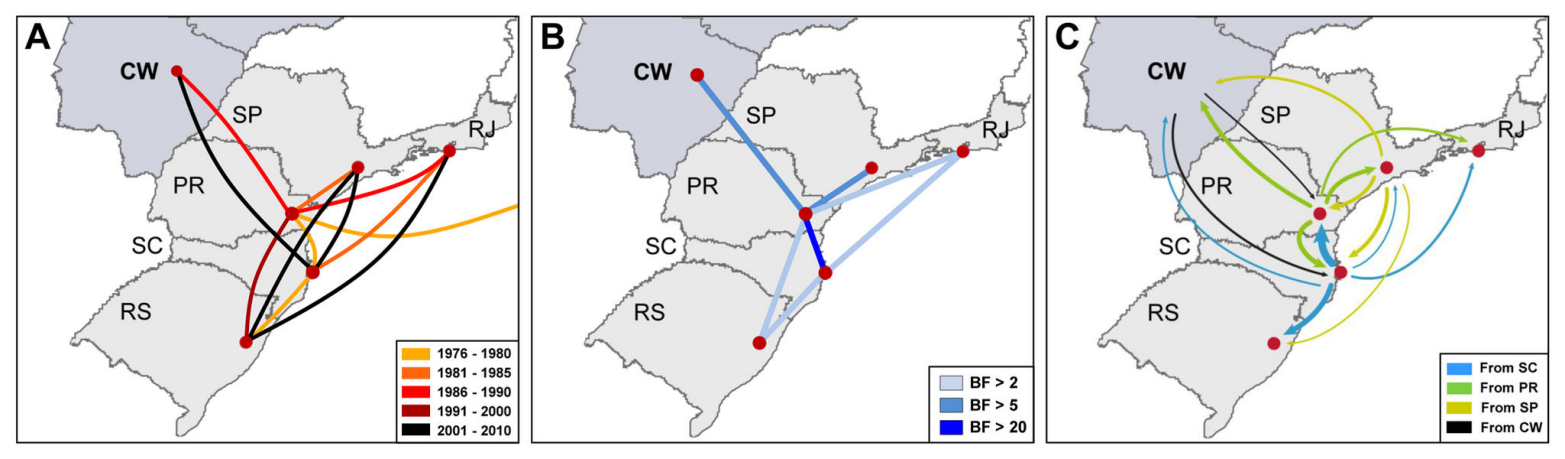
Spatiotemporal dynamics of HIV-1 C_BR-1_ clade dissemination in Brazil. (A) Viral dispersal pattern between 1976 and 2010. Lines between locations represent branches in the Bayesian MCC tree along which location transitions occurs. The yellow-black color gradient of lines informs the date of the earliest viral migrations among each pair of locations. (B) Bayes factor (BF) test for significant non-zero rates. Only rates supported by a BF greater than 2 are indicated. The light-dark color gradient of lines informs the relative strength by which the rates are supported (weak-strong). The maps are based on satellite pictures made available in Google^™^ Earth (http://earth.google.com). (C) Major estimated viral transitions rates (*q*) as measured by the APES program. The arrows were colored according to the source region and the width is proportional to *q*. All *q* lower than 1.0 were excluded for clarity. RS (Rio Grande do Sul), SC (Santa Catarina), PR (Paraná), RJ (Rio de Janeiro), SP (São Paulo) and CW (Central-west Region).

Reconstruction of viral migrations across time with the BEAST program revealed a rapid dissemination of the virus across Brazilian regions (Figure 6A). Between 1976 and 1980, the virus moves from PR to SC and from there to RS. During the 1980s, the virus migrates from PR to the southeast and central-west regions and from SC to RJ. In the following years, migration events from PR to RS, from SC to SP and the central-west region, and from RS to the southeast region were also registered. The Bayes factor (BF) tests for significant non-zero rates indicate well-supported rates (BF > 5) between PR/SC, PR/SP and PR/central-west region, and weakly supported rates (BF > 2) between PR/RS, PR/RJ, SC/RS and SC/RJ (Figure 6B). Viral movements among Brazilian localities were also estimated with the APE package. By comparing the marginal likelihoods for each model, we found that the asymmetric one outperformed the other two models (Figure S3 and Table S4). Confirming previous analysis, PR and SC states displayed the most representative estimates of viral exchange and also acted as the most important hubs of spread to the southeast and central-west regions (Figure 6C and S4). This analysis further suggests that SP could be a secondary hub of viral dissemination to the south and central-west regions (Figure 6C and S4); while RS, RJ and the central-west regions came out as receiving ends (i.e., a sink), having few lineages moving to other states (Figure 6C and S4).

### Human mobility and spread of the HIV-1 C_BR-I_ clade

**Figure 7:**
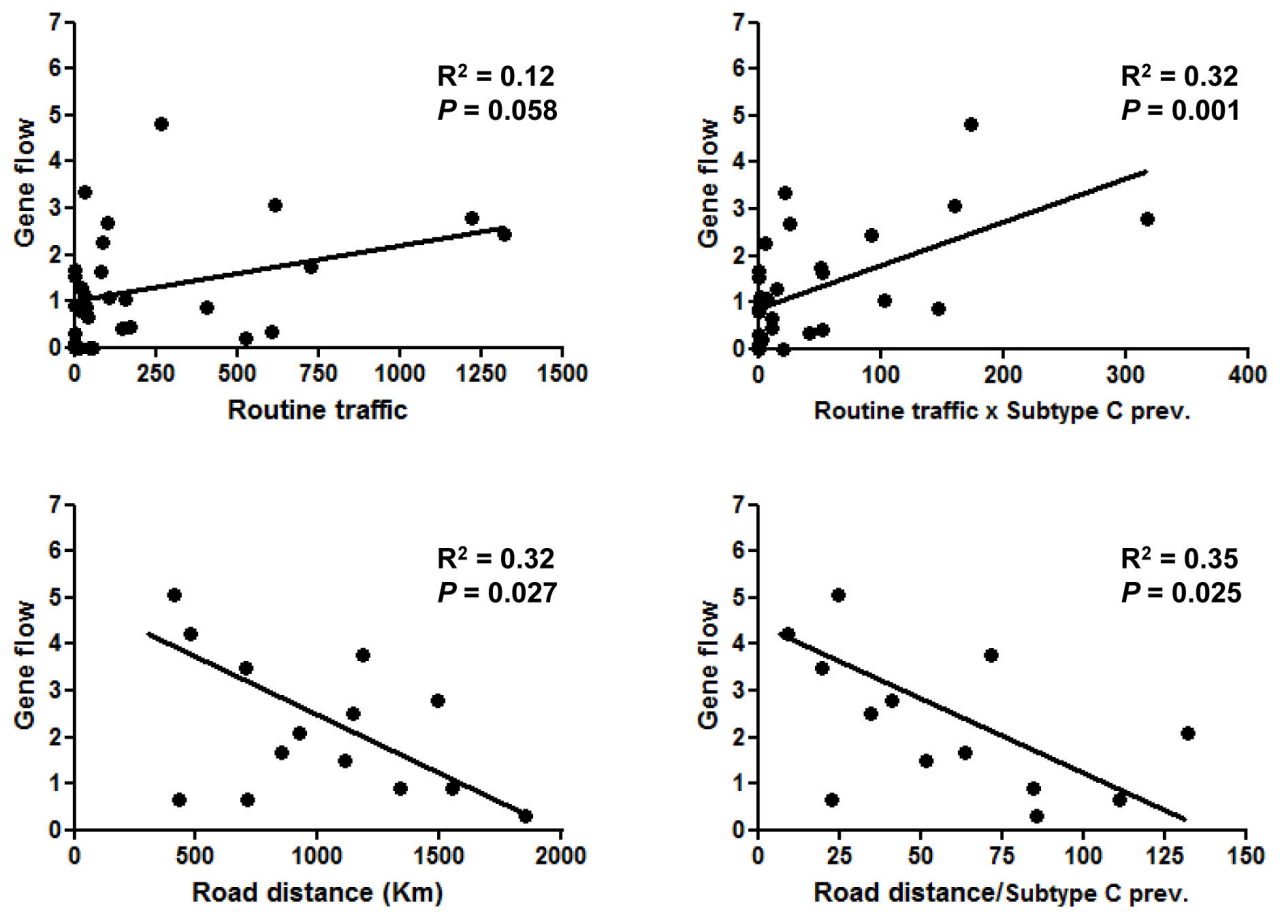
Association between viral migration rates and human mobility data. Viral transition rates (*q*, gene flow) were plotted against: (A) routine traffic ([people × trip]/1,000); (B) routine traffic multiplied by the HIV-1 subtype C prevalence in the site of origin; (C) road distances between state’s capitals (the capital of Goiás state was used as reference for the central-west region); and (D) road distances divided by the average subtype C prevalence in the corresponding states. The linear regression line is shown in each graph. The *P*-value and r squared (R^2^) from correlations are indicated in each plot.

To test the relevance of human mobility on the dissemination of HIV-1 subtype C epidemic in Brazil, viral transition rates estimated from the APE package were fitted to the routine travel and road distances between Brazilian localities from the south, southeast and central-west regions (Table S5). We found that viral movement between localities trend to be positively correlated with routine traffic among them (Figure 7A), although such a correlation became statistically significant (*P* < 0.05) only after the routine traffic was adjusted according to the estimated prevalence of subtype C in the state of origin (Figure 7B). We also found a significant negative correlation between viral transition rates and road distance, irrespective of the adjustment to the prevalence of subtype C in the state of origin (Figure 7C and 7D). Despite the statistical significance, the correlation coefficients obtained for all associations were low (R^2^ < 0.4).

## Discussion

The results presented here confirm the hypothesis that the major HIV-1 subtype C lineage circulating in Brazil (C_BR-I_) originated in east Africa [4,7,8] and further show that this Brazilian lineage belongs to the previously called C_EA_ clade [26]. It has been estimated that the C_EA_ clade comprises 100% of the HIV-1 subtype C sequences from Burundi, 97% from Uganda, 64% from Kenya, 61% from Ethiopia, 49% from Tanzania and 9% from central African countries; while it is absent or extremely rare in southern Africa [26]. Among all African countries where the C_EA_ clade circulates, Burundi is the most probable source of the Brazilian C_BR-I_ lineage.

The median *T*_mrca_ of the C_BR-I_ clade was previously estimated at 1980-1983 [4], 1977-1980 [8] and 1962-1977 [5]. The two datasets here analyzed (C_EA+BR-I_ and C_BI+BR-I_) consistently traced the origin of the C_BR-I_ clade back to the middle 1970s (1974-1976) and situate the median upper and lower limits for subtype C introduction in Brazil at 1972 and 1976, respectively. This time-frame coincides with the onset date of other major country-specific C_EA_ subclades detected in Ethiopia, Kenya, Tanzania and Uganda [26]. Interestingly, the estimated dissemination of the C_EA_ clade from Burundi to other east African countries and Brazil overlapped with the first major civil conflict that took place in Burundi in 1972 and generated around 300,000 refugees [49]. This large human migration flow exiting Burundi may have played a crucial role in the regional and international dissemination of the C_EA_ clade. The exact route of migration of the C_EA_ clade from Burundi to Brazil, however, remains unclear. It has been suggested that the United Kingdom (UK) may have acted as a staging post in the dissemination of subtype C between east Africa and Brazil [8]; but another study found no evidence of viral flow from the UK to Brazil, only from east Africa and Brazil to the UK [5].

Our phylogeographic reconstruction places the root of the C_BR-I_ clade in the state of PR with highest probability (PSP = 0.83), in agreement with previous studies [5,25]. By the early 1980s, the C_BR-I_ clade was already disseminated to the other southern Brazilian states, while between 1983 and 1988 the virus reached the southeast and the central-west regions. Despite a long-standing presence of the C_BR-I_ clade in all Brazilian regions, the final outcome of this HIV-1 clade across localities vary greatly. While subtype C accounts for 20-80% of HIV-1 infections in the southern states, the prevalence of this subtype remains ≤ 10% in the southeast and central-west regions. A recent study also showed an important expansion of HIV-1 subtype C infections amongst pregnant women from interior cities from the GO state, but not among those from the metropolitan area [16]. Thus, factors other than viral genetic characteristic and/or timing of viral introduction have shaped the expansion rate of the C_BR-I_ clade in different Brazilian regions. We propose that difference in the HIV transmission networks operating across localities may have contributed to such a heterogeneous spatial distribution pattern.

The states of PR and SC seems to be the main hubs of dissemination of the C_BR-I_ clade, exporting viruses to the other states. Estimation of viral movements with the APE package suggests that SP could be a secondary hub of viral dissemination sending viruses to the south and central-west regions; although this epidemiological link was not confirmed in the analysis with the BEAST program. Despite the high prevalence of subtype C and the large number of HIV cases in RS, this southernmost state seems to have a marginal role in the dissemination of the C_BR-I_ clade, sending only a few viral lineages to SC, SP and RJ. The results presented here point to a partially isolated subtype C epidemic in RS, consistent with our previous findings [25]. A large proportion (78%) of subtype C infections in RS appeared to be the result of the *in situ* dissemination of a single local sublineage (C_BR-RS_) that probably emerged at around 1980 and is mostly restricted to that area. Some highly supported geographic-specific monophyletic clades of small size were also identified in RJ, SP and the central-west region, revealing the existence of local transmission networks operating outside the southern region.

Routine travel and spatial accessibility among Brazilian regions has been pointed as possible driving forces of subtype C dissemination [5,25] and our results are fully consistent with this model. Viral exchanges between Brazilian localities increase as the routine traffic increases and the road distance (accessibility) decreases. The seeding of subtype C in the central-west region mainly from PR is also in line with the recent human migration in that direction, due to soybean plantation and similar agricultural activities. The HIV-1 subtype C prevalence in each locality seems to be another important factor to explain the rates of viral migration. The overall low prevalence of subtype C in SP (<10%) and RJ (<1%), for example, may explain the low viral exchange between both states despite their close geographical proximity and high routine traffic. By contrast, accessibility, human mobility and subtype C prevalence cannot explain the low level of viral migration from RS to SC. Indeed, the low correlation coefficients observed (R^2^ < 0.4) indicate that additional factors also have influenced the viral dissemination process.

While the C_BR-I_ lineage was the only subtype C clade detected in the southern and central-west Brazilian regions, five additional subtype C introductions were detected in the southeast region, particularly in the state of RJ. Although those five subtype C viruses may have been acquired locally, there is no evidence that they have become widely disseminated in the country as they were represented by only one individual each. Other studies have also identified the circulation of HIV-1 variants of African origin in the states of RJ and SP, including subtype D and the CRF02_AG [10,20,50,51,52]. These states host large international airports, ports and sociocultural and economic events, which create an excellent milieu for the introduction of new HIV-1 strains in the area. Of note, our phylogeographic analyses suggest that two of those five subtype C viruses introduced into RJ were probably imported from Burundi and/or Kenya. The identification of these additional introductions uncovers the existence of unrecognized transmission networks linking Brazil to east Africa.

The unequal number of sequences available from different countries and Brazilian regions can introduce large biases in phylogeographic reconstructions and influence the conclusions. Some of our key findings, however, were robust to the sampling scheme used here. Although most (39%) HIV-1 sequences of the C_EA_ clade were from Burundi, a putative epidemiological link between the Brazilian lineage C_BR-I_ and any other east African country could be easily established because most sequences from Ethiopia, Kenya, Tanzania and Uganda were distributed in well defined country-specific sub-clades. The clade C_BR-I_, however, was clearly placed among Burundian sequences and outside the major specific sub-clades from the other east African countries, thus supporting the Burundian origin of that major subtype C Brazilian lineage. Our study also indicates that PR was the most probable entrance point and one of the main hubs of dissemination of clade C_BR-I_ in Brazil despite the majority of subtype C Brazilian sequences within the major clade were from RS and SC.

In summary, the results presented here suggest that the HIV-1 subtype C epidemic spreading in most Brazilian states was initiated at around the middle 1970s by the introduction of a single founder strain originated in Burundi. Such a founder subtype C variant was probably introduced into PR and was rapidly disseminated to the other Brazilian states, originating the major C_BR-I_ clade. The states of PR and SC seem to be the most important hubs of the HIV-1 subtype C dissemination in Brazil. The explanation for the dissemination process of C_BR-I_ clade in Brazil is multifactorial and includes human mobility, accessibility, and local founder events among others. This study also identifies a continuous introduction of new HIV-1 subtype C variants of African origin into the RJ state. These results emphasize the importance of the continuous surveillance of HIV-1 subtype C genetic diversity to understand the dissemination dynamics of the C_BR-I_ clade at country level and for earliest detection of the introduction and dissemination of newly emerging subtype C viral clades in the Brazilian population.

## Supporting Information

Figure S1 **Substitution saturation and likelihood mapping analyses.** (A) Transition (blue line) and transversion (green line) versus divergence plot for the different HIV-1 subtype C pol datasets. (B) Percentage of dots plotted in each region of the map after likelihood mapping of 10,000 random quarters selected from the different HIV-1 subtype C pol datasets. Each dot represents the likelihoods of the three possible tree topologies for a set of four sequences (quartets) selected randomly from the dataset. The dots localized on the vertices, in the center and on the laterals represent the tree-like, the star-like and the network-like phylogenetic signals, respectively.
(PDF)

Figure S2 **ML tree of HIV-1 subtype C *pol* (~1,000pb) sequences from Brazil (*n* = 209) and South Africa (*n* = 1,031).** Branches of Brazilian sequences are represented in red. Those branches of South African sequences that were more closely related to the Brazilian ones and were selected for further phylogenetic analyses are indicated in green. For visual clarity, some Brazilian and South African clades were collapsed. The *aLRT* support values are indicated only at key nodes. The tree was rooted using HIV-1 subtype A1 and D reference sequences (gray branches). Horizontal branch lengths are drawn to scale with the bar at the bottom indicating nucleotide substitutions per site.
(PDF)

Figure S3 **Distribution of the likelihood for three distinct models of viral transition rates.** ER: model with equal rates among localities (black line). SYM: model with symmetric rates among localities (blue line). ARD: model with asymmetric rates among localities (red line).
(PDF)

Figure S4 **Estimated viral transition rates (*q*) to and from each locality.** All *q* lower than 0.5 were excluded for clarity. A – RS (Rio Grande do Sul, in red). B-SC (Santa *Catarina* , in blue). C-PR (Paraná, in green). D-SP (São Paulo, in yellow). E-RJ (Rio de Janeiro, in purple). F-CW (Central-west region, in black). The arrows width is proportional to *q* (available in Table S5).
(PDF)

Table S1 **HIV-1 C_AFR+BR_ dataset.**
(DOC)

Table S2 **HIV-1 C_EA+BR-I_ and C_BI+BR-I_ datasets.**
(DOC)

Table S3 **HIV-1 C_AFR+BR-II-VI_ dataset.**
(DOC)

Table S4 **Harmonic mean of Likelihoods for distinct models of viral transition rates.**
(DOC)

Table S5 **Viral transition rates, routine travels and road distances between localities.**
(DOC)
