# Supplementary figures and images for: Tracing the Origin and Northward Dissemination Dynamics of HIV-1 Subtype C in Brazil
Source: PLoS One. 2013 Sep 12;8(9):e74072. doi: 10.1371/journal.pone.0074072 (PMC3771961; doi:10.1371/journal.pone.0074072)

A

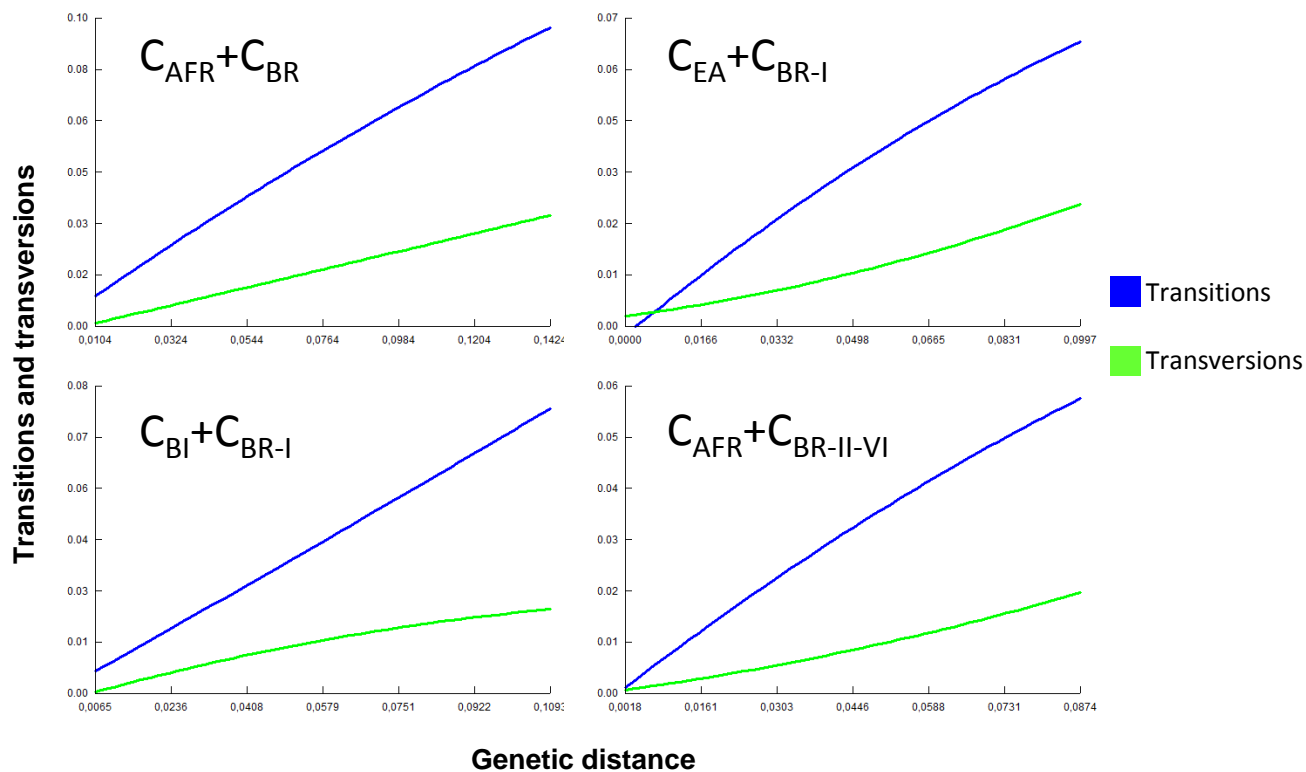

B

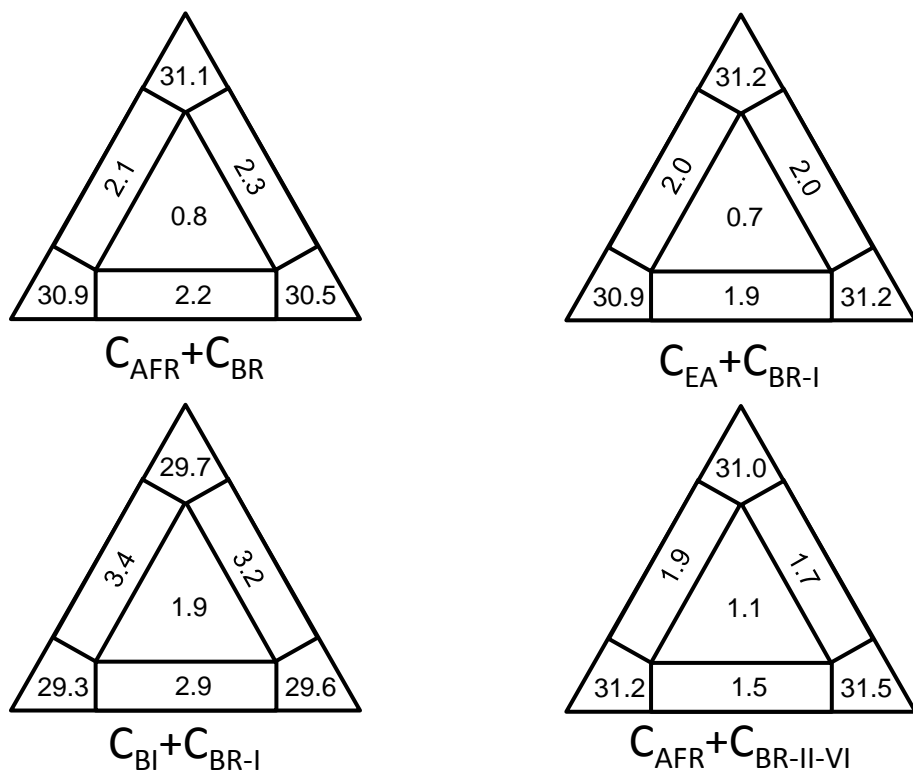

Supplement: Figure S1 — Substitution saturation and likelihood mapping analyses. (A) Transition (blue line) and transversion (green line) versus divergence plot for the different HIV-1 subtype C pol datasets. (B) Percentage of dots plotted in each region of the map after likelihood mapping of 10,000 random quarters selected from the different HIV-1 subtype C pol datasets. Each dot represents the likelihoods of the three possible tree topologies for a set of four sequences (quartets) selected randomly from the dataset. The dots localized on the vertices, in the center and on the laterals represent the tree-like, the star-like and the network-like phylogenetic signals, respectively. (PDF) [file pone.0074072.s001.pdf]

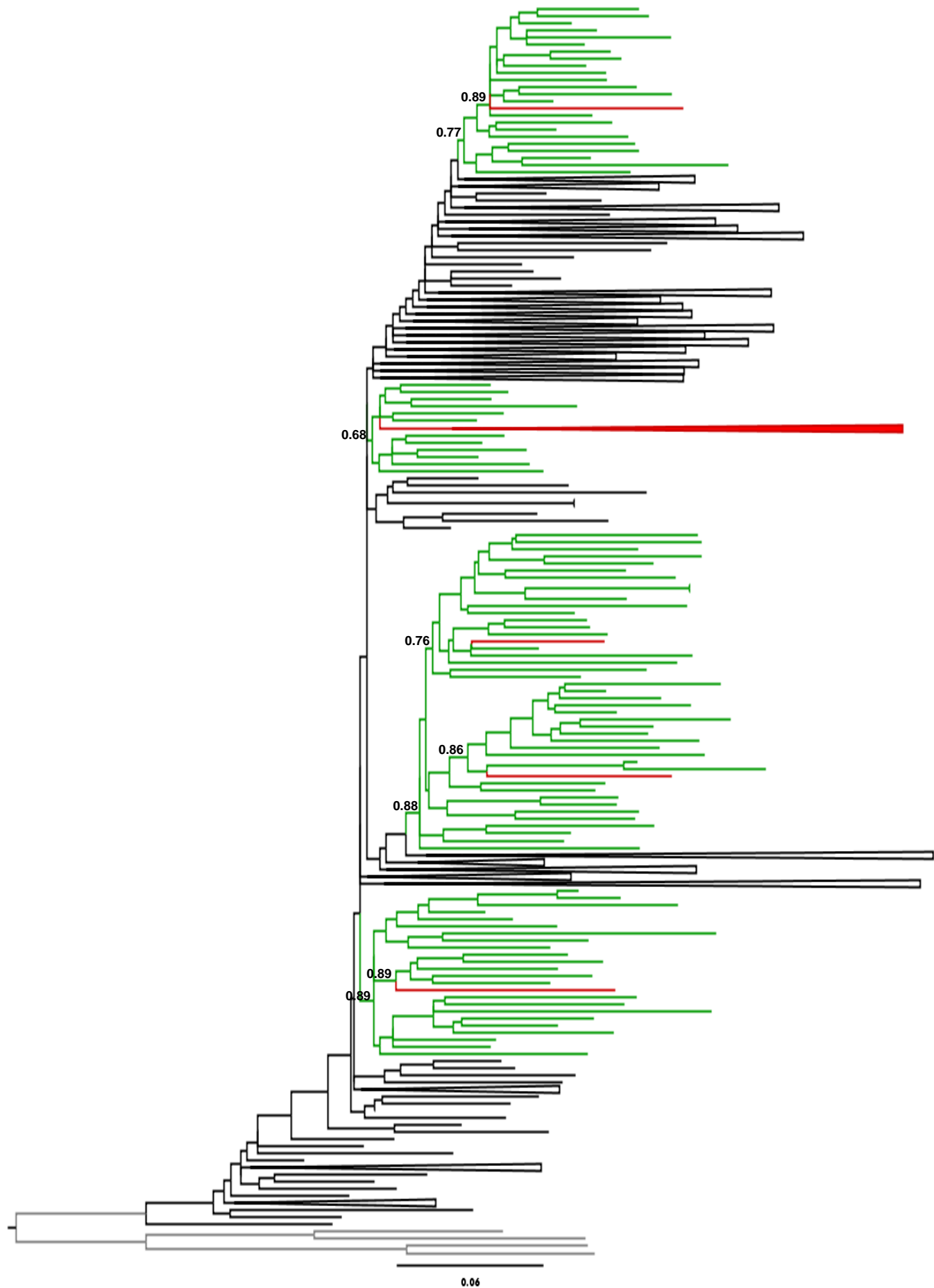

Supplement: Figure S2 — ML tree of HIV-1 subtype C pol (~1,000pb) sequences from Brazil (n = 209) and South Africa (n = 1,031). Branches of Brazilian sequences are represented in red. Those branches of South African sequences that were more closely related to the Brazilian ones and were selected for further phylogenetic analyses are indicated in green. For visual clarity, some Brazilian and South African clades were collapsed. The aLRT support values are indicated only at key nodes. The tree was rooted using HIV-1 subtype A1 and D reference sequences (gray branches). Horizontal branch lengths are drawn to scale with the bar at the bottom indicating nucleotide substitutions per site. (PDF) [file pone.0074072.s002.pdf]

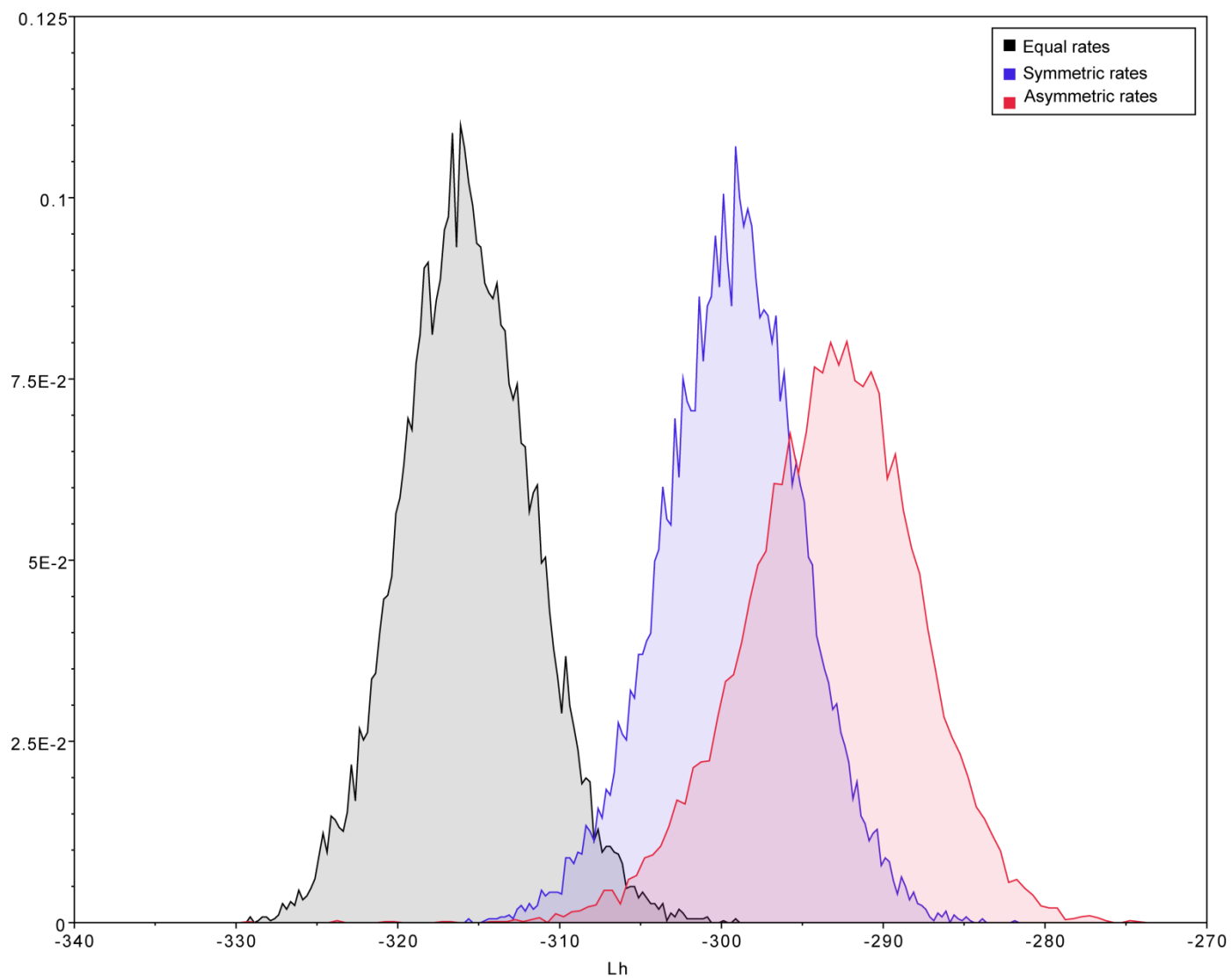

Supplement: Figure S3 — Distribution of the likelihood for three distinct models of viral transition rates. ER: model with equal rates among localities (black line). SYM: model with symmetric rates among localities (blue line). ARD: model with asymmetric rates among localities (red line). (PDF) [file pone.0074072.s003.pdf]

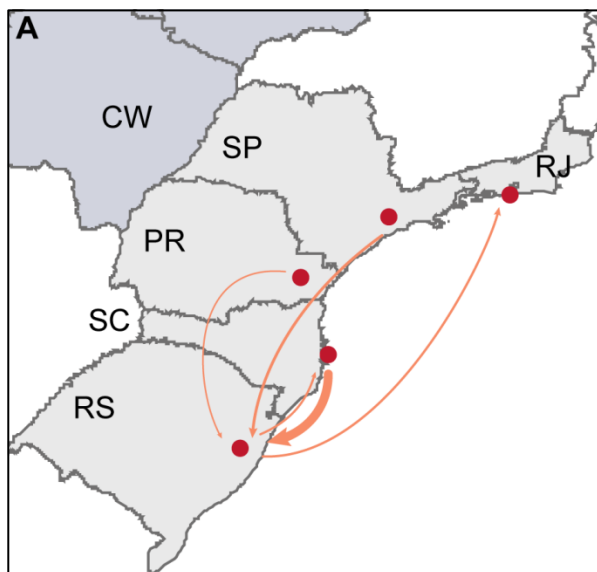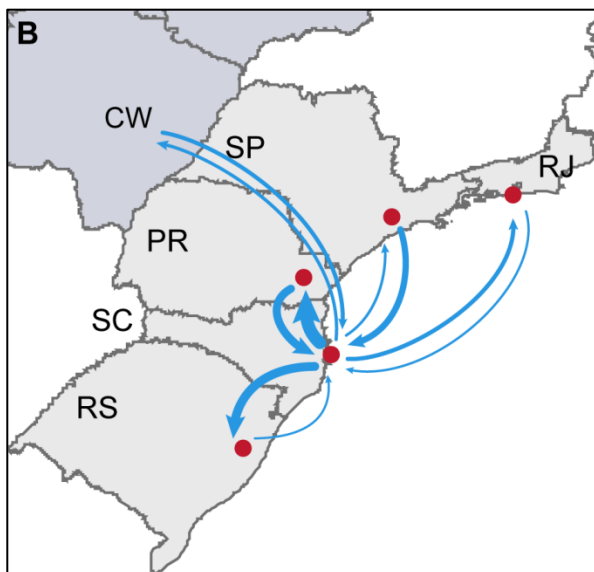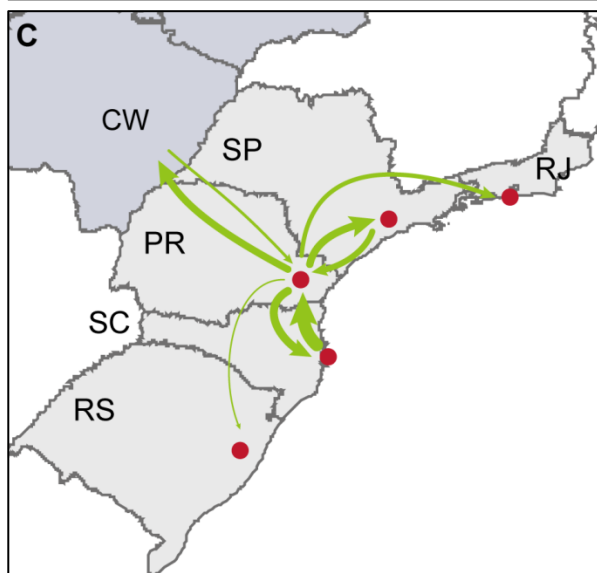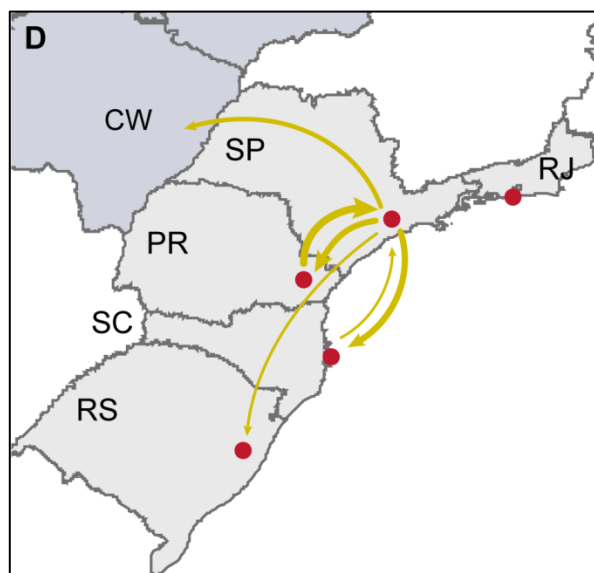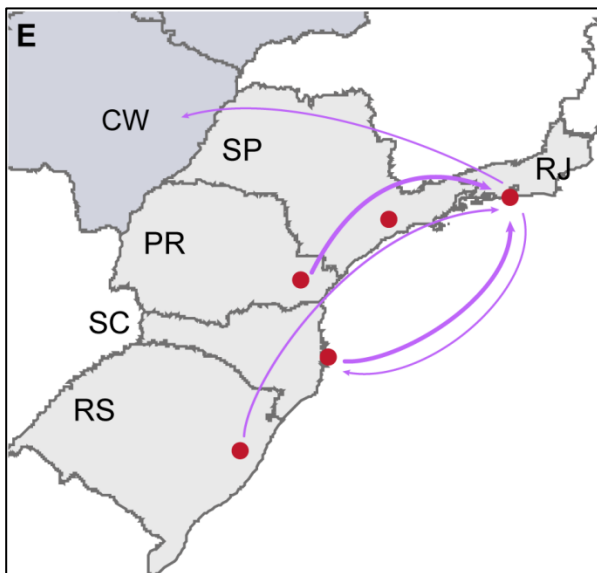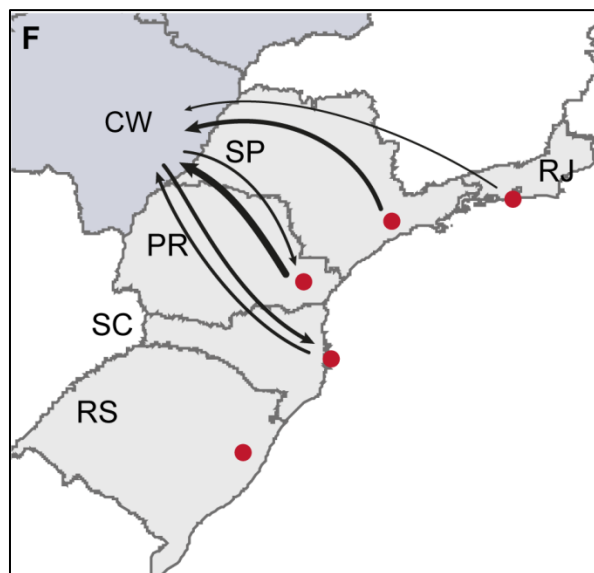

Supplement: Figure S4 — Estimated viral transition rates (q) to and from each locality. All q lower than 0.5 were excluded for clarity. A – RS (Rio Grande do Sul, in red). B-SC (Santa Catarina , in blue). C-PR (Paraná, in green). D-SP (São Paulo, in yellow). E-RJ (Rio de Janeiro, in purple). F-CW (Central-west region, in black). The arrows width is proportional to q (available in Table S5). (PDF) [file pone.0074072.s004.pdf]
