# Supplementary material for: Tracing the Origin and Northward Dissemination Dynamics of HIV-1 Subtype C in Brazil
Source: PLoS One. 2013 Sep 12;8(9):e74072. doi: 10.1371/journal.pone.0074072 (PMC3771961; doi:10.1371/journal.pone.0074072)
Supplement: Table S1 — HIV-1 CAFR+BR dataset. (DOC) [file pone.0074072.s005.doc]

**Table S1. HIV-1 C_AFR+BR_ dataset.**

| **Region** | **Country/State** | ***N*** | **Sampling date** |
| --- | --- | --- | --- |
| Brazil (South) | PR | 39 | 2001-2007 |
|  | RS | 55 | 1992-2006 |
|  | SC | 41 | 2008-2009 |
| Brazil (Southeast) | RJ | 32 | 2003-2011 |
|  | SP | 18 | 2004-2009 |
| Brazil (Central-west) | GO/MT/MS | 24 | 2003-2010 |
| Central Africa | Angola | 31 | 2001-2010 |
|  | Democratic Republic of Congo | 22 | 2002-2007 |
| Southern Africa | Botswana | 70 | 2001 |
|  | Malawi | 46 | 2002 |
|  | Mozambique | 101 | 2002-2004 |
|  | South Africa | 1,031 | 1999-2009 |
|  | Zambia | 150 | 1998-2008 |
|  | Zimbabwe | 178 | 2007 |
| East Africa | Burundi | 92 | 2002 |
|  | Ethiopia | 82 | 1986-2003 |
|  | Kenya | 39 | 1991-2007 |
|  | Tanzania | 81 | 1997-2009 |
|  | Uganda | 38 | 1990-2010 |
