# Supplementary material for: Tracing the Origin and Northward Dissemination Dynamics of HIV-1 Subtype C in Brazil
Source: PLoS One. 2013 Sep 12;8(9):e74072. doi: 10.1371/journal.pone.0074072 (PMC3771961; doi:10.1371/journal.pone.0074072)
Supplement: Table S2 — HIV-1 CEA+BR-I and CBI+BR-I datasets. (DOC) [file pone.0074072.s006.doc]

**Table S2. HIV-1 C_EA+BR-I_ and C_BI+BR-I_ datasets.**

| **Dataset** | **Region** | **Country/State** | ***N*** | **Sampling date** |
| --- | --- | --- | --- | --- |
| C_EA+BR-I_ | Brazil (South) | PR | 10 | 2001-2006 |
|  |  | RS | 10 | 1992-2006 |
|  |  | SC | 10 | 2008-2009 |
|  | East Africa | Burundi | 92 | 2002 |
|  |  | Ethiopia | 47 | 2002-2003 |
|  |  | Kenya | 24 | 2004-2007 |
|  |  | Tanzania | 40 | 2005-2009 |
|  |  | Uganda | 33 | 1990-2010 |
| C_BI+BR-I_ | Brazil (South) | PR | 39 | 2001-2007 |
|  |  | RS | 55 | 1992-2006 |
|  |  | SC | 41 | 2008-2009 |
|  | Brazil (Southeast) | RJ | 27 | 2003-2011 |
|  |  | SP | 18 | 2004-2009 |
|  | Brazil (Central-west) | GO/MT/MS | 24 | 2003-2010 |
|  | East Africa | Burundi | 10 | 2002 |
