# Supplementary material for: Tracing the Origin and Northward Dissemination Dynamics of HIV-1 Subtype C in Brazil
Source: PLoS One. 2013 Sep 12;8(9):e74072. doi: 10.1371/journal.pone.0074072 (PMC3771961; doi:10.1371/journal.pone.0074072)
Supplement: Table S3 — HIV-1 CAFR+BR-II-VI dataset. (DOC) [file pone.0074072.s007.doc]

**Table S3. HIV-1 C_AFR+BR-II-VI_ dataset.**

| **Region** | **Country/State** | ***N**** | **Sampling date** |
| --- | --- | --- | --- |
| Brazil (Southeast) | RJ | 5 | 2006-2011 |
| Central Africa | Angola | 2 (2/0) | 2001-2010 |
| East Africa | Burundi | 9 (3/6) | 2002 |
|  | Ethiopia | 3 (0/3) | 1996-2003 |
|  | Kenya | 15 (14/1) | 2004-2007 |
|  | Tanzania | 2 (2/0) | 2005 |
|  | Uganda | 1 (1/0) | 1997 |
| Southern Africa | Botswana | 4 (0/4) | 2000-2001 |
|  | Malawi | 4 (0/4) | 2000-2009 |
|  | Mozambique | 1 (1/0) | 2002 |
|  | South Africa | 32 (19/13) | 2000-2009 |
|  | Zambia | 15 (2/13) | 1989-2008 |
|  | Zimbabwe | 2 (2/0) | 2007 |
| Western Africa | Senegal | 2 (0/2) | 1990-2003 |
| Asia | China | 1 (0/1) | 2004 |
|  | India | 1 (0/1) | 2000 |
| Europe | Austria | 1 (0/1) | 2004 |
|  | Sweden | 1 (0/1) | 2004 |

*In parenthesis is indicated the number of non-Brazilian reference sequences selected using ML analysis/BLAST.
