# Supplementary material for: Tracing the Origin and Northward Dissemination Dynamics of HIV-1 Subtype C in Brazil
Source: PLoS One. 2013 Sep 12;8(9):e74072. doi: 10.1371/journal.pone.0074072 (PMC3771961; doi:10.1371/journal.pone.0074072)
Supplement: Table S4 — Harmonic mean of Likelihoods for distinct models of viral transition rates. (DOC) [file pone.0074072.s008.doc]

**Table S4.** Differences among harmonic mean of Likelihoods for three distinct transition models.

| **Model** | **ln P(model | data)** | **S.E.** | **Equal rates** | **Symmetric rates** | **Asymmetric rates** |
| --- | --- | --- | --- | --- | --- |
| Equal rates | -321,307 | +/- 0.078 | - | -15,145 | -18,754 |
| Symmetric rates | -306,162 | +/- 0.106 | 15,145 | - | -3,609 |
| Asymmetric rates | -302,553 | +/- 0.152 | 18,754 | 3,609 | - |
