# Supplementary material for: Tracing the Origin and Northward Dissemination Dynamics of HIV-1 Subtype C in Brazil
Source: PLoS One. 2013 Sep 12;8(9):e74072. doi: 10.1371/journal.pone.0074072 (PMC3771961; doi:10.1371/journal.pone.0074072)
Supplement: Table S5 — Viral transition rates, routine travels and road distances between localities. (DOC) [file pone.0074072.s009.doc]

**Table S5.** Viral transition rates, routine travels and road distances between localities.

| **Localities^a^** | **Transition rate (*q*)^b^** | **Routine travels ^c^**  **(people × trip)/1,000** | **Road distances (Km)^d^** |
| --- | --- | --- | --- |
| RJ to SC | 0.88 | 39 | 1144 |
| PR to SC | 3.06 | 618 | 300 |
| SP to SC | 2.43 | 1320 | 705 |
| RS to SC | 0.86 | 409 | 476 |
| CW to SC | 1.52 | 0 | 1493 |
| SC to RJ | 1.64 | 80 | 1144 |
| PR to RJ | 1.67 | 0 | 852 |
| SP to RJ | 0.46 | 171 | 429 |
| RS to RJ | 0.90 | 4 | 1553 |
| CW to RJ | 0.10 | 1 | 1338 |
| SC to PR | 4.82 | 267 | 300 |
| RJ to PR | 0.00 | 19 | 852 |
| SP to PR | 2.27 | 86 | 408 |
| RS to PR | 0.00 | 59 | 711 |
| CW to PR | 1.07 | 107 | 1186 |
| SC to SP | 1.05 | 158 | 705 |
| RJ to SP | 0.21 | 526 | 429 |
| PR to SP | 2.79 | 1223 | 408 |
| RS to SP | 0.41 | 147 | 1109 |
| CW to SP | 0.34 | 608 | 926 |
| SC to RS | 3.35 | 34 | 476 |
| RJ to RS | 0.00 | 45 | 1553 |
| PR to RS | 0.64 | 42 | 711 |
| SP to RS | 1.10 | 33 | 1109 |
| CW to RS | 0.00 | 0 | 1847 |
| SC to CW | 1.27 | 24 | 1493 |
| RJ to CW | 0.79 | 17 | 1338 |
| PR to CW | 2.70 | 102 | 1186 |
| SP to CW | 1.74 | 726 | 926 |
| RS to CW | 0.32 | 0 | 1847 |

^a^ RS (Rio Grande do Sul), SC (Santa Catarina), PR (Paraná), RJ (Rio de Janeiro), SP (São Paulo) and CW (Central-west Region). ^b^ Mean transition rates (*q*) estimated for the posterior set of 18,000 trees using the APE package. ^c^ Routine traffic amongst states according to estimations of the Public Ministry of Tourism (<http://www.dadosefatos.turismo.gov.br/export/sites/default/dadosefatos/demanda_turistica/domestica/downloads_domestica/Relatxrio_Executivo_Tur_Dom_2007.pdf>) for the year 2007. ^d^ Road distances between state’s capitals (the capital of Goiás state was used as reference for the central-west region).
